# Comparison of methohexital and propofol as induction agents for evaluation of laryngeal function in healthy dogs

**DOI:** 10.1111/vsu.13110

**Published:** 2018-10-27

**Authors:** Mikala B. Brown, Danielle R. Dugat, Shane D. Lyon, Laura A. Nafe, Mark E. Payton, Sarah K. Peakheart, Rebecca S. Salazar

**Affiliations:** ^1^ Department of Veterinary Clinical Sciences Oklahoma State University Stillwater Oklahoma; ^2^ Department of Statistics Oklahoma State University Stillwater Oklahoma; ^3^ Blue Pearl Veterinary Emergency and Specialty Hospital Spring Texas

## Abstract

**Objective:**

To determine the influence of propofol or methohexital, with and without doxapram, on the examination of laryngeal function in dogs.

**Study design:**

Experimental study.

**Animals:**

Forty healthy dogs randomly assigned to 4 groups: propofol with saline (n = 10), propofol with doxapram (n = 10), methohexital with saline (n = 10), or methohexital with doxapram (n = 10).

**Methods:**

Propofol and methohexital were administered to effect. Investigators examined laryngeal function (initial) simultaneously with video laryngoscopy. Doxapram or saline was administered, and laryngeal function was reevaluated (second). Laryngeal motion, quality of laryngeal exposure, and the degree of swallowing, laryngospasm, and jaw tone were scored at each evaluation. Adverse events were recorded. Initial and second videos were evaluated by a masked observer, and still images obtained from both evaluations were evaluated for change in rima glottidis size by 2 masked observers.

**Results:**

Administration of doxapram and saline was delayed with propofol (*P* = .001). Laryngeal function did not differ between dogs receiving propofol or methohexital, irrespective of doxapram administration. Doxapram improved breathing scores in both groups (*P* < .001). Jaw tone increased with propofol during the second evaluation (*P* = .049). Swallowing was more prevalent at initial examination (*P* = .020). Methohexital resulted in an increased heart rate (*P* < .001) compared with propofol. Twenty‐five percent of dogs receiving methohexital developed seizure‐like activity (n = 5/20).

**Conclusion:**

Evaluation of laryngeal function did not differ between healthy dogs anesthetized with propofol or methohexital. Methohexital provided shorter examination times with less jaw tone but was associated with adverse events.

**Clinical significance:**

This study provides evidence to recommend propofol over methohexital as an induction agent for laryngeal function examination.

## 1 INTRODUCTION

Laryngeal paralysis is a condition commonly diagnosed in older, large, and giant‐breed dogs.^1^, ^2^ Laryngeal paralysis is characterized by decreased or absent functional arytenoid motion, which can result in upper airway obstruction. Clinical signs range from mild to severe, including mild inspiratory stridor to life‐threatening dyspnea and collapse.^1^, ^2^, ^3^ These clinical signs are often dictated by progression of the disease from unilateral to bilateral arytenoid paralysis.^1^, ^2^ Exercise intolerance, heat intolerance, and aspiration pneumonia may also be present. Laryngeal paralysis can be congenital or acquired and is caused by degeneration or damage to the recurrent laryngeal nerve.^1^, ^2^ Laryngeal paralysis is thought to be a component of a more generalized polyneuropathy in many dogs. Paraparesis and altered esophageal function can be present concurrently with laryngeal paralysis in dogs affected with this polyneuropathy.^1^

A tentative diagnosis of laryngeal paralysis is based on the dog's history and physical examination findings. The diagnosis is confirmed with direct laryngeal examination, oral laryngoscopy, transnasal laryngoscopy, or echolaryngography.^2^, ^4^, ^5^, ^6^, ^7^, ^8^, ^9^ The objective of laryngeal examination is to evaluate arytenoid abduction on inspiration. Absent or asymmetric motion is consistent with a diagnosis of laryngeal paralysis.^4^, ^6^ Echolaryngography is not recommended because of its low sensitivity and specificity.^9^ Transnasal laryngoscopy can be performed in awake sedated dogs but does not improve diagnostic accuracy.^9^ The preferred diagnostic method is to examine the larynx under oral laryngoscopy, which requires a light plane of anesthesia. The ideal induction agent provides a smooth induction and recovery while achieving a depth of anesthesia resulting in minimal jaw tone without substantial impairment of normal arytenoid function.^4^, ^5^, ^6^, ^10^ Anesthetic induction agents that can impair normal function of arytenoid cartilages may lead to false positive diagnoses of laryngeal paralysis.

Anesthetic protocols that have been evaluated for laryngeal evaluation include propofol, thiopental, ketamine, alfaxalone, and various combinations of these agents.^4^, ^5^, ^6^, ^7^ Each of these agents may result in dose‐dependent respiratory depression, affecting the examination. Jackson et al^5^ found that dogs anesthetized with thiopental displayed greater arytenoid motion near recovery than any other protocol used. Smalle et al^7^ concluded that propofol was superior because it resulted in shorter examination times without affecting the evaluation of laryngeal function compared with alfaxalone and thiopentone (thiopental). Despite any disparity in preferential induction agents, thiopental is no longer available for clinical use in the United States; propofol is currently the most commonly used anesthetic agent for laryngeal examination. However, propofol can induce substantial respiratory depression or apnea, subsequently impairing laryngeal function and examination.^11^ The addition of ketamine did not palliate the respiratory depression induced by propofol in a study of dogs undergoing evaluation of laryngeal motion.^4^ Smalle et al^7^ found that lower dosage rates of propofol played an important role in providing a more rapid identification of laryngeal function. Doxapram, a respiratory stimulant, has been found helpful in overcoming the apnea and/or shallow breathing often induced by current anesthetic protocols.^10^, ^12^ An important finding was that this agent rapidly increased respiration rates in normal dogs and dogs affected with laryngeal paralysis. This rapid increase in respiration rate also led to paradoxical motion of the arytenoid cartilages in dogs affected with laryngeal paralysis.^10^ Laryngeal motion in normal dogs did not appear to change, but the percentage change in glottal gap area was increased.^10^

Identifying a new protocol for laryngeal examination may improve the quality of evaluation and accuracy of diagnosis of laryngeal paralysis. Methohexital is a rapidly acting barbiturate anesthetic agent, similar to thiopental.^13^, ^14^ It produces a short period of anesthesia as a single agent. Potential adverse effects of methohexital include tachycardia, seizurelike events, and necrosis with perivascular administration.^15^ No studies have evaluated the quality of laryngeal motion in dogs anesthetized with methohexital.

The objective of this study was to determine the influence of propofol or methohexital with or without doxapram on the examination of laryngeal function in healthy dogs. Our primary hypothesis was that no difference in evaluation of laryngeal function in healthy dogs would be found between methohexital and propofol, as assessed by a prior published scale for laryngeal function assessment.^6^ Our secondary hypothesis was that there would be no appreciable difference in evaluation of laryngeal function in dogs receiving doxapram, irrespective of the induction agent.

## 2 MATERIALS AND METHODS

### 2.1 Animals

Forty healthy young adult dogs were recruited among dogs presented to the shelter program at the Oklahoma State University Center for Veterinary Health Sciences from local shelters for routine spay or neuter between September and December 2016. The experimental protocol was approved by the university's institutional animal care and use committee, and each participating shelter signed an informed consent for enrollment of their dogs in the study. Inclusion criteria included the absence of respiratory abnormalities, normal physical examination findings, and a body weight greater than 5.0 kg.

### 2.2 Experimental design

Each dog was randomly assigned to 1 of 2 groups, a control group (group P) and an experimental group (group M), by using a computerized randomization protocol (Excel; Microsoft, Redmond, Washington). Group P consisted of 20 dogs in which layngeal evaluation was performed under propofol‐induced anesthesia. Half of these dogs (n = 10) were randomized to receive doxapram for respiratory stimulation while half (n = 10) were given a saline placebo. Group M consisted of 20 dogs in which laryngeal evaluation was performed under methohexital‐induced anesthesia. Dogs in this group were similarly randomly assigned to receive doxapram (n = 10) to stimulate respiratory function or saline placebo (n = 10).

Prior to induction, a complete physical examination was performed on each dog by the same individual (MBB). An intravenous catheter was placed in a cephalic vein. The induction protocol excluded any premedications from being administered. Dogs in group P were scheduled to receive up to a 6.0‐mg/kg^16^ IV dose of propofol, given to effect, reducing the potential for apnea or an excessively deep plane of anesthesia. Dogs in group M were scheduled to receive up to an 11.0‐mg/kg^16^ IV dose of methohexital, with half given steadily as a bolus and the remaining volume administered to effect. Investigators performing the oral laryngeal evaluations (DRD and MBB) were not masked to the induction agent being administered. Doxapram was administered at a dose of 2.2 mg/kg IV,^16^ or saline was administered as an equal volume to allow the investigators to be masked to which dogs received doxapram.

Appropriate plane of anesthesia was confirmed by loss of jaw tone allowing for adequate visualization of the larynx. An experienced diplomate of the American College of Veterinary Internal Medicine (ACVIM; LAN) performed video laryngoscopy using a 30 °, 2.7‐mm rigid cystoscope (Karl Storz Veterinary Endoscopy America, Goleta, California). Direct visual observation of the larynx was also performed concurrently by an experienced diplomate of the American College of Veterinary Surgeons (DRD) and a surgical intern (MBB). A tertiary investigator noted whether the dog was undergoing inspiration or expiration along with depth of respiration to allow documentation of any paradoxical arytenoid motion. Two video and visual examinations were obtained for each dog. The first examination, referred to as *initial*, was performed at administration of propofol or methohexital, and the second examination, referred to as *second*, was performed after administration of doxapram or saline.

Data were recorded for each dog for initial and second laryngeal evaluations by using a modification of a previously published scale and included breathing score (0‐3); laryngeal motion score (0‐2); quality of visualization/laryngeal exposure (poor, moderate, excellent); and the presence or absence of swallowing (0‐2), laryngospasm (0‐2), and jaw tone (0‐3; Table 1).^6^ Heart rate and electrocardiogram were monitored and recorded throughout the examination. Any adverse events pertaining to induction or recovery were recorded. The length of each procedure was recorded, with intermediary time points corresponding to time from start of induction to evaluation and administration of doxapram or saline specifically noted. After the laryngeal examination was complete, each dog underwent the previously scheduled elective gonadectomy.

**Table 1:** Criteria and grading scale used during visual laryngeal examination^a^

Laryngeal examination response	Score	Definition
Breathing score		
	0	No spontaneous respiration
	1	Shallow respiration, slow respiratory rate, weak attempt
	2	Moderate respiration, rate, and attempt
	3	Deep respiration, normal respiratory rate, strong attempt
Laryngeal motion		
	0	Abnormal/no arytenoid movement
	1	Weak/inconsistent arytenoid movement
	2	Strong/consistent arytenoid movement
Jaw tone		
	0	No jaw tone
	1	Slight jaw tone
	2	Moderate jaw tone
	3	Excessive jaw tone
Laryngeal exposure		
	P	Poor
	M	Moderate
	E	Excellent
Laryngospasm		
	0	Absent
	1	Present
Swallowing		
	0	Absent
	1	Present

a Each dog was scored on the basis of the definition of that response provided.

Each video examination was evaluated at a later date by a masked observer (SDL), who is a diplomate of the ACVIM. Each video was evaluated for degree of laryngeal motion and presence or absence of laryngospasm and swallowing by the same scores noted above.

An objective evaluation of the change in rima glottidis between inspiration and expiration was performed individually by 2 investigators (DRD and SDL) using still images from the obtained videos. The height and width of the rima glottidis was measured on 6 images, 3 at maximal abduction and 3 at maximal adduction, in ImageJ 1.50i (National Institutes of Health, http://imagej.nih.gov/ij). The ratio of each value at maximal abduction and adduction was calculated. The 3 ratio measurements were then averaged to allow for normalization of data. The difference in the ratio at maximal adduction prior to doxapram or saline administration and the ratio at maximal abduction postadministration was calculated. The difference in ratio of maximal adduction compared with maximal abduction was also calculated before and after doxapram or saline administration to account for the effects of doxapram and time.

### 2.3 Statistical analysis

All statistical analyses were conducted in SAS version 9.4 (SAS, Cary, North Carolina). A power analysis was performed in R software (https://www.r-project.org/) with still image variables of maximal abduction and adduction of the larynx, yielding the ability to detect a standard deviation difference with 88% power. The variables of interest were evaluated for normality by using Kolmogorov‐Smirnoff tests, with no compelling evidence to suspect nonnormality. Analysis of variance assuming a factorial arrangement in a completely randomized design was used to evaluate the initial and second differences between induction agent administered, observer, and doxapram administration. Simple effects of each factor given the other factors (eg, effect of doxapram given observer and induction agent) were evaluated by using planned comparisons within the ANOVA model. Contingency tables and χ^2^ analyses were used to assess differences in the observers’ responses for examinations to breathing, jaw tone, exposure, laryngeal function, swallowing, and laryngeal spasm (both initial and second examinations). An independent *t* test was performed on adverse events that occurred with administration of the induction agents. Fisher's exact test was used to evaluate for the rate of seizure activity between induction agent groups. Statistical significance was set at *P* < .05.

## 3 RESULTS

Forty dogs were enrolled in the study. Ages for dogs varied between 3 months and 5 years (mean, 1.6 years). There were 27 males and 13 females with no known history or physical examination findings consistent with respiratory disease or laryngeal paralysis. The body weight of the dogs ranged from 5.0 to 29.5 kg, with a mean body weight of 15.4 kg.

Half of the calculated total dose of methohexital was administered as the initial steady bolus over a mean of 22 seconds (range, 10‐40). The total volume administered was then titrated to achieve the appropriate anesthetic plane for examination. Propofol was administered slowly and to effect over a mean of 208 seconds (range, 60‐420). Mean doses administered for methohexital were 7.4 mg/kg (range, 4.9‐10.6) and for propofol were 6.8 mg/kg (range, 2.3‐12.1). Time from beginning of first injection to administration of the second injection (doxapram or saline) was longer in group P (263.2 ± 67.9 seconds) compared with group M (177.7 ± 67.9, *P* = .001). Time from end of first injection to administration of second injection of saline for group P (120.111 ± 25.6 seconds) was not different from group M (115.0 ± 28.3 seconds; *P* = .8607). Time from end of first injection to administration of second injection of doxapram for group P (71.7 ± 10.2 seconds) was not different from group M (113.6 ± 10.5 seconds, *P* = .1455).

### 3.1 Visual laryngoscopy

There was no difference between observers (DRD and MBB) performing visual laryngoscopy in determination of laryngeal function, either before (initial) or after (second) administration of doxapram (initial, *P* = .65; second, *P* = .72; Table 2). There was no difference in laryngeal function between groups P and M before administration of doxapram (initial, *P* = .22) or after administration of doxapram (second, *P* = .22).

**Table 2:** Degree of laryngeal function^a^

Laryngeal function score response	Observer	Method of induction, No. of dogs (%)					
		Initial P	Initial M	Second P + S	Second P + D	Second M + S	Second M + D
0	DRD	6 (31.5)	10 (50)	0 (0)	0 (0)	3 (30)	0 (0)
	MBB	7 (35)	10 (55.5)	1 (11.1)	0 (0)	0 (0)	0 (0)
1	DRD	4 (21)	5 (25)	4 (44.4)	2 (20)	3 (30)	3 (33.3)
	MBB	8 (40)	6 (33.3)	6 (60)	4 (40)	5 (55.6)	3 (37.5)
2	DRD	9 (47.3)	5 (25)	5 (55.5)	8 (80)	4 (40)	6 (66.)
	MBB	5 (25)	2 (11.1)	3 (30)	6 (60)	4 (44.4)	5 (62.5)
Total responses	DRD	19	20	9	10	10	9
	MBB	20	18	10	10	0	8
*P* value		.65		.72			

D, doxapram; M, methohexital; P, propofol; S, saline.

a Evaluated by direct observers (DRD and MBB) during initial and second evaluations of group P and group M performed. Each number represents the frequency of dogs (%) in which the degree of laryngeal function was identified. Laryngeal motion was characterized with 0 = abnormal/no arytenoid movement, 1 = weak/inconsistent arytenoid movement, or 2 = strong/consistent arytenoid movement. The first observer (DRD) was not available for evaluation of 1 dog in the P group, yielding 39 dogs in total that were evaluated. The second observer (MBB) was absent for evaluation of 2 dogs in the M group, yielding 38 dogs in total that were evaluated. Both observers (DRD and MBB) did not record 1 second evaluation for a dog in the M + D group because this dog vomited and developed signs of seizure‐like activity.

There was no difference between direct observers (DRD and MBB) in evaluation of breathing score (initial, *P* = .159; second, *P* = .966), laryngeal exposure (*P* = .30), and the presence or absence of swallowing in the second phase (*P* = .0545), laryngeal spasm (initial, *P* = .0531; second, *P* = .2403), or jaw tone (initial, *P* = .23; second, *P* = .469; Tables 3, 4, 5, 6, 7). There was a difference between direct observers in identification of swallowing during the initial phase (*P* = .0156; Table 5).

**Table 3:** Breathing scores^a^

Breathing score response	Observer	Method of induction, No. of dogs (%)					
		Initial P	Initial M	Second P + S	Second P + D	Second M + S	Second M + D
0	DRD	1 (5.2)	1 (5)	0 (0)	0 (0)	0 (0)	0 (0)
	MBB	0 (0)	3 (16.7)	0 (0)	0 (0)	0 (0)	0 (0)
1	DRD	8 (42.1)	11 (55)	3 (33.3)	0 (0)	5 (50)	0 (0)
	MBB	12 (60)	12 (66.7)	4 (40)	0 (0)	4 (44.4)	0 (0)
2	DRD	8 (42.1)	5 (25)	3 (33.3)	2 (20)	3 (30)	4 (40)
	MBB	8 (40)	2 (11.1)	3 (30)	2 (20)	5 (55.5)	3 (33.3)
3	DRD	2 (10.5)	2 (10)	3 (33.3)	8 (80)	2 (20)	6 (60)
	MBB	0 (0)	1 (5.6)	3 (30)	8 (80)	0 (0)	6 (66.6)
Total responses	DRD	19	20	9	10	10	10
	MBB	20	18	10	10	9	9
*P* value		.159		.966			

D, doxapram; M, methohexital; P, propofol; S, saline.

a Assigned by direct observers (DRD and MBB) during initial and second evaluations of group P and group M. Each number represents the frequency of dogs (%)in which the depth of breathing was scored. Breathing was scored as 0 = no spontaneous respirations, 1 = shallow respiration, slow respiratory rate, weak attempt, 2 = moderate respiration rate and attempt, and 3 = deep respiration, normal respiratory rate, and strong attempt. The first observer (DRD) was not available for evaluation of 1 dog in the P group, yielding 39 dogs in total that were evaluated. The second observer (MBB) was absent for evaluation of 2 dogs in the M group, yielding 38 dogs in total that were evaluated.

**Table 4:** Degree of laryngeal exposure^a^

Degree of laryngeal exposure	Observer	Method of induction, No. of dogs (%)	
		Propofol	Methohexital
Poor	DRD	0 (0)	0 (0)
	MBB	0 (0)	0 (0)
Moderate	DRD	0 (0)	0 (0)
	MBB	0 (0)	2 (11.1)
Excellent	DRD	19 (100)	20 (100)
	MBB	20 (100)	16 (88.9)
Total responses	DRD	19	20
	MBB	20	18
*P* value		.30	

a Identified by direct observers (DRD and MBB) in evaluations of group P (propofol) and group M (methohexital). Each number represents the frequency of dogs, combined with a percentage, in which the degree of laryngeal exposure was identified during examination. Exposure was documented as poor, moderate, or excellent. The first observer (DRD) was not available for evaluation of 1 dog in the propofol group, yielding 39 dogs in total that were evaluated. The second observer (MBB) was absent for evaluation of 2 dogs in the methohexital group, yielding 38 dogs in total that were evaluated.

**Table 5:** Swallowing response^a^

Swallowing response	Observer	Method of induction, No. of dogs (%)					
		Initial P	Initial M	Second P + S	Second P + D	Second M + S	Second M + D
0	DRD	11 (55)	19 (95)	9 (100)	10 (100)	10 (100)	10 (100)
	MBB	11 (55)	16 (88.9)	9 (90)	9 (90)	9 (100)	7 (77.8)
1	DRD	8 (42.1)	1 (5)	0 (0)	0 (0)	0 (0)	0 (0)
	MBB	9 (45)	2 (11.1)	1 (10)	1 (10)	0 (0)	2 (22.2)
Total responses	DRD	19	20	9	10	10	10
	MBB	20	18	10	10	9	9
*P* value		.0156		.0545			

D, doxapram; M, methohexital; P, propofol; S, saline.

a Identified by direct observers (DRD and MBB) in initial and second evaluations of group P and group M. Swallowing was scored as 0 = absent or 1 = present. The first observer (DRD) was not available for evaluation of 1 dog in the P group, yielding 39 dogs in total that were evaluated. The second observer (MBB) was absent for evaluation of 2 dogs in the M group, yielding 38 dogs in total that were evaluated.

**Table 6:** Laryngospasm^a^

Laryngospasm response	Observer	Method of induction, No. of dogs (%)					
		Initial P	Initial M	Second P + S	Second P + D	Second M + S	Second M + D
0	DRD	17 (89.5)	19 (95)	9 (100)	10 (100)	10 (100)	10 (100)
	MBB	13 (65)	16 (88.9)	10 (100)	10 (100)	9 (100)	7 (77.8)
1	DRD	2 (10.5)	1 (5)	0 (0)	0 (0)	0 (0)	0 (0)
	MBB	7 (35)	2 (11.1)	0 (0)	0 (0)	0 (0)	2 (22.2)
Total responses	DRD	19	20	9	10	10	10
	MBB	20	18	10	10	9	9
*P* value		.0531		.2403			

D, doxapram; M, methohexital; P, propofol; S, saline.

a Identified by direct observers (DRD and MBB) during initial and second evaluations of groups P and M. Laryngospasm was scored as 0 = present or 1 = absent. The first observer (DRD) was not available for evaluation of 1 dog in the P group, yielding 39 dogs in total that were evaluated. The second observer (MBB) was absent for evaluation of 2 dogs in the M group, yielding 38 dogs in total that were evaluated.

**Table 7:** Summarization of the degree of jaw tone^a^

Jaw tone response	Observer	Method of induction, No. of dogs (%)					
		Initial P	Initial M	Second P + S	Second P + D	Second M + S	Second M + D
0	DRD	9 (47.3)	14 (70)	6 (66.7)	4 (40)	7 (70)	7 (77.8)
	MBB	12 (60)	15 (83.3)	6 (60)	5 (50)	8 (88.9)	7 (87.5)
1	DRD	9 (47.4)	6 (30)	3 (33.3)	6 (60)	3 (30)	2 (22.2)
	MBB	7 (35)	3 (16.7)	2 (20)	5 (50)	1 (11.1)	1 (12.5)
2	DRD	1 (5.3)	0 (0)	0 (0)	0 (0)	0 (0)	0 (0)
	MBB	1 (5)	0 (0)	2 (2)	0 (0)	0 (0)	0 (0)
3	DRD	0 (0)	0 (0)	0 (0)	0 (0)	0 (0)	0 (0)
	MBB	0 (0)	0 (0)	0 (0)	0 (0)	0 (0)	0 (0)
Total responses	DRD	19	20	9	10	10	9
	MBB	20	18	10	10	9	8
*P* value		.23		.469			

D, doxapram; M, methohexital; P, propofol; S, saline.

a Identified between direct observers (DRD and MBB) in initial and second evaluations of groups P and M. Jaw tone was scored as 0 = no jaw tone, 1 = slight jaw tone, 2 = moderate jaw tone, and 3 = excessive jaw tone. The first observer (DRD) was not available for evaluation of 1 dog in the P group, yielding 39 dogs in total that were evaluated. The second observer (MBB) was absent for evaluation of 2 dogs in the M group, yielding 38 dogs in total that were evaluated. Both observers (DRD and MBB) did not record 1 second evaluation for a dog in the M + D group because this dog vomited and developed signs of seizure‐like activity.

There was no difference in initial breathing scores (*P* = .214) between methohexital and propofol; however, there was a difference observed in second breathing scores (*P* < .001). Post hoc analysis revealed improved breathing scores in group M dogs that received methohexital and doxapram vs methohexital and saline, in group P dogs that received propofol and doxapram vs propofol and saline, and in group P dogs that received propofol and doxapram vs group M dogs that received methohexital and saline (*P* < .001). These improved breathing scores did not result in paradoxical movement of the arytenoid cartilages. There was no difference in jaw tone between groups during initial evaluation (*P* = .22). There were more patients in group P with increased jaw tone during the second evaluation compared with group M (*P* = .049). There was no difference in laryngeal exposure between groups P and M (*P* = .377). There were more patients in group P with swallowing present during the initial examination (*P* = .020), although a difference was not noted for the second examination (*P* = .534). There was no difference in the presence of laryngospasm between groups P and M (initial, *P* = .056; second, *P* = .534).

### 3.2 Image evaluation from video laryngoscopy

There was a difference in ratio measurements between observers (DRD and SDL) in the evaluation of the difference between initial narrow (the narrowest width before saline administration) and second wide (the widest width after saline administration) of both groups P (*P* < .001) and M (*P* = .0065) without doxapram administration. In this evaluation, 1 observer (DRD) measured a smaller difference in the change from initial narrow to second wide (group P: −0.17047, SE 0.036516; group M: −0.18247, SE 0.042292) compared with the other observer (SDL; group P: −0.40676, SE 0.056999; group M: −0.29260, SE 0.056638). There was also a difference in laryngeal function, identified by the change in rima glottidis size from initial narrow to second wide, seen by 1 observer (DRD) in group P between dogs receiving doxapram (−0.40676, SE 0.054549) and those receiving saline (−0.17047, SE 0.036516; *P* = .028). No further difference in laryngeal function was seen with administration of doxapram or between agents administered.

Evaluation of video laryngoscopy by the masked observer (SDL) revealed a difference in the presence of swallowing (initial, *P* = .009; second, *P* = .014), laryngospasm (initial, *P* < .001; second, *P* < .001), and laryngeal function postdoxapram or saline administration (second, *P* = .001) for groups P and M combined compared with the findings of the direct observers (DRD and MBB). No difference between blinded and nonblinded observers was seen with initial evaluation of laryngeal function (*P* = .118).

### 3.3 Adverse events

Adverse events identified in group M were tachycardia, seizure‐like activity, vomiting and regurgitation. Mean heart rate of dogs in group M prior to administration of methohexital was 132 beats/min (range, 110‐187), and mean heart rate after administration of methohexital was 229 beats/min (range, 190‐256). Dogs receiving propofol (group P) had a lower mean heart rate after administration (145 beats/min; range, 80‐210) compared with dogs receiving methohexital (*P* < .001). Seizure‐like activity was seen in 25% of the methohexital cases (n = 5/20); 2 episodes occurred concurrently with vomiting (n = 1) and regurgitation (n = 1). There was a difference between seizure‐like activity in group M vs group P (*P* = .047). All seizure‐like episodes occurred as the dogs were returning to a lighter plane of anesthesia at completion of the laryngeal function examination; 1 dog began having seizure‐like activity directly after intubation. After identification of seizure‐like activity in 3 of the first 4 dogs receiving methohexital, the anesthetic protocol was modified to include an additional bolus of methohexital if dogs were returning to a light plane of anesthesia and required intubation for their elective gonadectomy procedure after laryngeal function examination. After altering the protocol, 2 of the remaining 16 dogs still developed seizurelike signs. No adverse events were seen in dogs in group P.

## 4 DISCUSSION

The main finding of this study is that laryngeal function did not differ in dogs anesthetized with propofol or methohexital, prompting us to accept our primary hypothesis. These results are in line with the recent conclusions of Smalle et al,^7^ finding propofol superior to thiopentone, also a barbiturate, for evaluation of initial laryngeal function. Our results are also in line with those of Jackson et al,^5^ who found thiopental superior for evaluating laryngeal function, but evaluation of initial laryngeal function did not differ between thiopental and propofol.

Swallowing, laryngospasm, and laryngeal function differed when assessed by the masked observer (SDL) compared with direct observers (DRD and MBB). This difference may reflect the use of videos for evaluation by the masked observer, or potential bias by the direct observers, aware of the agents used. Video evaluation removes knowledge of respiratory status (presence of apnea), depth of respiration, and phase of respiration, which clearly could inhibit the ability of the masked observer correctly to identify adequate laryngeal function without paradoxical motion. It is also possible that the masked observer could pause the video and evaluate at leisure, which may improve the identification of occasional swallow or laryngospasm events. The difference in laryngeal function is more difficult to explain. The masked observer (SDL) appreciated more instances of “no function” in the second examinations compared with the direct observers. Laryngospasm or flutter may have been observed by the direct observers and qualified as brief function, whereas the masked observer had more time to evaluate the video and the opportunity to rewind to confirm weak or brief function. Some dogs who received doxapram maintained extremely exaggerated laryngeal function in which the larynx remained maximally abducted. The direct observers could identify these episodes because they correlated with exaggerated breathing, whereas the masked observer would not be able to recognize this change.

The use of video laryngoscopy compared with direct observation of laryngeal function warrants additional study. The authors believe that the direct observers’ evaluation should serve as gold standard when masked observers are unable to detect laryngeal function in dogs identified as functional through direct assessment. Indeed, direct observers can identify subtle changes in respiratory rate, identify the depth of respiration, know the pattern of laryngeal motion as it correlates to phase of respiration, and identify the plane of anesthesia. Each of these components of an examination is important to evaluate a dog successfully for laryngeal paralysis.

Doxapram did not influence laryngeal function in this study, irrespective of induction agent used, prompting us to accept our secondary hypothesis. However, administration of doxapram increased breathing scores. Such increase can enhance the confidence of an experienced observer assessing laryngeal function, but breathing can also result in paradoxical motion of the larynx. This effect could have contributed to the variability of laryngeal function scores in the current study.

Examination consistently took longer when dogs received propofol rather than methohexital. Achieving an adequate plane of induction was delayed with propofol, which pushed back initial evaluation times as well as the time to administration of the second injection (doxapram or saline). After adequate anesthetic plane was reached, no difference was detected between agents in times between the end of the first injection and the start of the second injection. Lack of adequate anesthetic plane also required administration of a greater volume of propofol than anticipated in 5 dogs. Likewise, dogs receiving propofol consistently had more jaw tone and swallowing after initial drug administration, resulting in a longer time required to achieve the appropriate plane of anesthesia. This finding was expected because propofol administration can result in apnea if administered too fast. Therefore, slow administration of propofol is preferred to achieve an adequate plane of anesthesia to evaluate laryngeal function with the dog sedate enough to allow for adequate visualization.^5^, ^7^, ^17^ Premedication agents were not used in this study to prevent potential for increased sedation and depressed laryngeal function in the dogs. The lack of premedication resulted in the administration of higher doses of propofol and therefore, a deeper plane of anesthesia. This factor did not affect dogs receiving methohexital as an induction agent.

Evaluation of still images and the difference in rima glottidis size between the initial and second phases supported the absence of difference in methohexital and propofol for functional laryngeal evaluation. However, minor differences were identified between the 2 observers (DRD and SDL). One observer (SDL) identified a larger difference in the ratios obtained for the narrowest rima glottidis at the initial phase and the widest rima glottidis at the second phase compared with the other observer (DRD). This difference could reflect the difficulty in identifying landmarks in some images. Portions of the ventral floor of the larynx were sometimes cut off during recording of videos, complicating height measurements and associated mean ratio differences. These subtle differences in still image measurements highlight the value of visual examination to identify absolute change in rima glottidis size.

Adverse events were more severe in dogs receiving methohexital compared with those receiving propofol. Seizure‐like activity occurred in 25% of dogs as they approached a lighter plane of methohexital anesthesia. The majority of these complications occurred early in the study (3 of the first 4 dogs). The seizure‐like activity ceased with additional administration of methohexital. After noticing the consistent timing of seizure‐like activity in the first 3 events, the study protocol was modified with a second bolus of methohexital administered before the animal reached a light plane of anesthesia. This altered protocol suppressed the onset of seizure‐like signs in all but 2 of the remaining study animals. This side effect of methohexital is important to note because some dogs concurrently vomited. Vomiting and regurgitation can result in aspiration pneumonia, for which laryngeal paralysis dogs are already at risk.^1^, ^18^ No adverse events were documented in the propofol group. Despite reducing the occurrence of seizure‐like activity in most dogs after modification of the induction protocol for methohexital, the continued seizure‐like activity that occurred in 2 subsequent dogs and the lack of difference in laryngeal function identified does not warrant consideration for its use above propofol. Additional evaluation is required to evaluate whether the events seen at recovery from methohexital anesthesia are truly seizures or muscle fasiculations and seizure‐like activity. This may be especially important when considering its use in patients with a concurrent history of seizures. Tachycardia has also been reported as a common side effect during the induction of anesthesia with methohexital.^15^ Our study corroborated this finding, with the heart rate of dogs increasing initially and returning to a normal rate after 15‐20 minutes. This side effect should be considered when choosing an induction agent such as methohexital in a dog with concurrent cardiovascular pathology.

Limitations of the current study include a small sample, lack of a standardized scale measurement within the video laryngoscopy, and lack of masking direct observers to the anesthetic induction agent used. A larger sample may further isolate specific adverse events associated with methohexital; however, a power analysis conducted did yield the ability to detect a standard deviation difference with 88% power. The effects of doxapram may have been more apparent with a larger sample as well. Use of a scale within the field of view on laryngoscopy would have allowed direct measurements of the area of the rima glottidis rather than calculating ratios. The authors used a height:width ratio to standardize measurements, as previously described for objective evaluation of the change in the rima glottidis.^5^ Direct measurements may have provided a more accurate comparison. Failing to mask direct observers to the anesthetic induction agent used may have created bias. However, the agents differ in appearance (color), compromising the ability to mask observers to the drug while performing an adequate direct oral examination.

In conclusion, no difference was detected in evaluation of laryngeal function of dogs anesthetized with methohexital or propofol at a similar depth of anesthesia. Therefore, methohexital was not found to be a better induction agent to evaluate laryngeal function. Methohexital provided shorter examination times and decreased jaw tone compared with propofol but resulted in more adverse events. Administration of doxapram did not influence the evaluation of laryngeal function, irrespective of the induction agent. The results of this study provide evidence to support continued use of propofol as the preferred induction agent for laryngeal function examination.

## CONFLICT OF INTEREST

The authors declare no conflicts of interest related to this report.
